# Seliciclib: A New Treatment for Cushing's Disease?

**DOI:** 10.17925/EE.2023.20.1.4

**Published:** 2023-11-08

**Authors:** Eleni Armeni, Ashley Grossman

**Affiliations:** 1. Department of Endocrinology and NET Unit, Royal Free Hospital, London, UK; 2. Centre for Endocrinology, Barts and the London School of Medicine, Queen Mary University of London, London, UK; 3. Green Templeton College, University of Oxford, Oxford, UK

**Keywords:** Cushing's disease, cyclin-dependent kinase inhibitor p27, cyclin E, seliciclib, pituitary-targeting agent, R-roscovitine

## Abstract

Previous studies have suggested that corticotroph tumours are associated with the overexpression of cyclin E and that the inactivation of cyclin-dependent kinases, which activate cyclin E, may have antisecretory and antiproliferative effects. Seliciclib, also known as R-roscovitine, is a pituitary-targeting agent shown to inhibit the growth of corticotroph tumour cells via cyclin E and retinoblastoma protein-mediated pathways. A recent study investigated the role of seliciclib in regulating biochemical parameters in a small number of patients with Cushing's disease, providing preliminary data on its possible therapeutic effectiveness in treating this disorder.

Cushing's disease (CD), or pituitary-dependent Cushing's syndrome, is almost always caused by corticotroph tumours, a type of pituitary neuroendocrine tumour, which overproduces adrenocorticotrophic hormone (ACTH), ultimately leading to hypercortisolism and its associated clinical consequences, including increased mortality.^1^ The complications associated with Cushing's syndrome must be minimized through effective treatment, ideally by rapidly normalizing hypercortisolaemia.^1^ The most effective treatment is transsphenoidal surgery, a procedure designed to remove the pituitary tumour, which, in expert hands, leads to a rapid lowering of cortisol levels and a period of hypocortisolaemia, with the gradual recovery of the normal corticotroph axis in 80–90% of patients.^2–4^ However, there is a notable failure rate of primary surgery and even repeat surgery, with a significant proportion of apparently ‘cured’ patients showing recurrence of their disease, such that some 30% of patients will require additional therapy.^2–4^ Various forms of radiotherapy can be used to treat CD, but these treatments are generally rather slow in onset and may require temporary medical treatment, usually with adrenostatic agents such as metyrapone and/or ketoconazole and, more recently, with levoketoconazole or osilodrostat.^4^ Bilateral adrenalectomy can also rapidly control hypercortisolaemia, but this may be followed by the development of a corticotroph tumour leading to Nelson's syndrome.^5^ The latter is considered to occur in only 20–25% of patients following bilateral adrenalectomy; however, a recent consensus has indicated tumour progression in approximately 40% of patients,^5^ and this figure may be even higher, with a significant proportion of patients not being controlled due to differences in functional capacity, size of pituitary tumour, and the age of the patient at the time of diagnosis.^6^

Therefore, there has been a search for pituitary-directed medical therapies directly targeting the corticotroph tumour to both lower ACTH levels and attenuate or entirely inhibit tumour growth. Currently, only two pituitary-directed agents are used treat corticotroph tumours: cabergoline and pasireotide. Cabergoline targets dopamine-2 receptors and is readily available; however it has demonstrated limited efficacy, and relapse is not uncommon.^7,8^ The somatostatin receptor ligand, pasireotide, has been shown to be active on somatostatin receptor subtype 5, a subtype which is characteristic of corticotroph tumours.^7,8^ However, its overall effectiveness is relatively limited: it tends to normalise cortisol levels mostly in just mild cases of CD and has marked hyperglycaemic effects, which can limit its use.^7^

Studies have been exploring the molecular biology of corticotroph tumours. Around 30% of such tumours have a pathogenic mutation of the *USP8* gene that leads to enhanced recycling of the epidermal growth factor receptor to the cell surface.^9^ Furthermore, there is evidence that the tyrosine kinase antagonist gefitinib can inhibit the downstream activation of the epidermal growth factor receptor in human corticotroph tumour cells of patients with CD, resulting in a reduction of ACTH secretion.^10^

In early studies, the cyclin-dependent kinase inhibitor p27 was shown to be downregulated in human pituitary tumours, and this was especially marked in corticotroph tumours.^11^ As p27 specifically interferes with cyclin-dependent kinase 2, which activates cyclin E, we were able to demonstrate the immunocytochemical overexpression of cyclin E in human corticotroph tumours.^12^ In a study investigating the expression of cyclin D1 and cyclin E among 95 human pituitary samples, comprising 20 nontumorous samples, 19 samples from patients with CD, 19 from patients with somatotroph tumours, 18 nonfunctioning pituitary neuroendocrine tumours, 9 aggressive pituitary tumours, 7 prolactinomas and 3 pituitary carcinomas, nuclear cyclin E was expressed statistically more frequently in the more aggressive compared with the less aggressive tumours and was particularly evident in corticotroph tumours from patients with CD.^12^

These findings suggested that the cyclin E/cyclin-dependent kinase-2 pathway would be especially susceptible to blockade in corticotroph tumours. Subsequently, studies in Shlomo Melmed's laboratory found that cyclin E overexpression was specifically associated with corticotroph tumour proliferation and ACTH secretion in both mice and zebrafish^13^ and that seliciclib, or R-roscovitine, a cyclin-dependent kinase inhibitor, could inhibit proliferation and ACTH secretion from human corticotroph tumours *in vitro*.^14^ Seliciclib has been demonstrated to disrupt the binding of the E2F transcription factor-1 and cyclin E to the promoter gene of pro-opiomelanocortin, inhibiting pro-opiomelanocortin expression in corticotroph cells.^13–15^

Based on these data, a prospective, open-l abel, phase II trial was recently published by Liu et al.^16^ This project initially evaluated the effect of seliciclib given at a dose of 400 mg twice daily for 4 days per week and for a total treatment duration of 4 weeks. The authors investigated the effect of the seliciclib treatment on 4 women with CD aged 43–66 years and with a disease duration of 0.25–9 years in a single-centre study (ClinicalTrials.gov identifier: NCT02160730) and, later, on 5 women with CD aged 19–58 years and with disease duration 0.5–10 years in a multi-centre study (ClinicalTrials.gov identifier: NCT03774446). Of these patients, two were treated with seliciclib while awaiting surgery, while seven were treated for persisting or recurrent CD. The multi-centre study was closed after the first 5 patients completed treatment rather than the planned sample size of 29 because of difficulties in patient recruitment due to the coronavirus disease 2019 pandemic.

Overall, mean urinary free cortisol (UFC) levels over 24 h decreased after the first week of treatment and rebounded from the second to the third week, lowering the UFC levels by 36% by the end of the study.^16^ The UFC level rebound was possibly related to the treatment being administered for only 4 days per week with a withdrawal of treatment for 3 days per week. No patient achieved normal UFC levels, but overall UFC levels fell by 42% in the 7 patients who completed the 4 weeks of treatment. Defining patients that achieved the most profound UFC reduction (>48%) as 'responders' and those that achieved a lesser reduction of UFC as 'nonresponders', there was a fall in plasma ACTH in the group of 'responders' only, indicating the ACTH-dependence of the fall in cortisol. Late-night salivary cortisol did not change.

Regarding adverse events, marked liver toxicity was reported in 3/9 patients (presenting as elevated liver enzymes) and grade 1 anaemia in 2/9 patients, which was possibly drug related. The effect of treatment on liver function resolved within 4 weeks of treatment discontinuation. The authors determined such adverse events to be related to the treatment dose based on their observations and clinical data.^16^

Therefore, what is the takeaway message from these findings? It is important to marry the molecular perturbations of pituitary tumours with putative therapies, as this is likely to be the way forward for future treatment paradigms. In the case of the study by Liu et al.,^16^ there is a clear proof of concept, and it is interesting that the overall fall in UFC level was found to be similar to that seen with pasireotide, albeit in a small number of patients. It does seem that hepatotoxicity is likely to limit any dose increase, and indeed, the authors are now exploring lower doses. However, any novel therapy is worth exploring for corticotroph tumours, which may become aggressive and are among the most common subtypes of (admittedly rare) metastatic pituitary tumours.^17^ It may be that new treatments are most advantageous for these more aggressive tumours, especially progressive tumours following bilateral adrenalectomy.
